# Workplace Anger Costs Women Irrespective of Race

**DOI:** 10.3389/fpsyg.2020.579884

**Published:** 2020-11-06

**Authors:** Christopher K. Marshburn, Kevin J. Cochran, Elinor Flynn, Linda J. Levine

**Affiliations:** ^1^Department of Psychology, University of Kentucky, Lexington, KY, United States; ^2^Department of Psychological Science, University of California, Irvine, Irvine, CA, United States; ^3^Stern School of Business, New York University, New York, NY, United States

**Keywords:** gender stereotypes, emotions, race, intersectionality, workplace

## Abstract

The current research investigated the role that a person’s race, gender, and emotional expressions play in workplace evaluations of their competence and status. Previous research demonstrates that women who express anger in the workplace are penalized, whereas men are not, and may even be rewarded. Workplace sanctions against angry women are often attributed to a backlash resulting from the violation of gender stereotypes. However, gender stereotypes may differ by race. The present study addressed this question using a between-subjects experimental design where participants (*N* = 630) read a vignette describing a new employee, which varied with respect to the employee’s race (White, Black, Asian, and Latino/a/x), gender (male and female), and a prior emotional response (anger and sadness). Participants then evaluated the employee’s competence and status. Findings revealed that men and women were both viewed as more competent when expressing anger relative to sadness, and this pattern did not differ across employee race. However, despite anger being associated with greater competence, women who violated stereotypes (i.e., expressed anger) were accorded lower status than stereotype-inconsistent (sad) men. Furthermore, exploratory analyses revealed that this pattern was consistent regardless of target and participant race. The current study replicates and extends previous research by employing an intersectional perspective and using a large, ethnically diverse sample to explore the interaction between gender and emotional expression on workplace evaluations across races.

## Introduction

Unreasonable bosses, missed promotions, lay-offs—these are just a few of the aspects of organizational life that can routinely elicit negative emotions from employees. Accordingly, anger, sadness, and other negative emotions are thought to be common affective experiences that impact the workplace (Barsade and Gibson, 2007; Gibson and Callister, 2010). Studying the expression of anger and sadness in organizations is important because these emotions differ in the extent to which they communicate dominance, which influences perceptions of status (Tiedens, 2001; Shields, 2002). Anger is intimidating, gains immediate compliance (Hochschild, 1990), and suggests to group members that the person deserves esteem (Tiedens, 2001). Sadness, in contrast, conveys weakness and incompetence (Tiedens, 2001). Yet, recent research shows that men and women who express negative emotions at work are evaluated differently (Rudman and Glick, 2001; Brescoll and Uhlmann, 2008). This work suggests that the status boost that results from expressing anger benefits only men (Brescoll and Uhlmann, 2008; Livingston et al., 2012). In part, such patterns might be explained by people’s stereotypic expectations for how men and women express emotion. For instance, some research demonstrates that people believe women are more likely than men to express sadness and less likely than men to express anger (Plant et al., 2000). Moreover, perceptions of dominance and affiliation influence how men and women’s emotions are perceived. Specifically, when women are perceived as less dominant than men, they are expected to express sadness relative to anger in emotionally distressing situations (Hess et al., 2005). Conversely, not only are men expected to show dominance and assertiveness generally, especially in competitive contexts, but some research suggests that they may face penalties at work if they are too agreeable (Judge et al., 2012). However, when women get angry, they are penalized in the form of being accorded lower status and rated as unlikeable when expressing anger or dominance because such expression violates the prescriptive stereotype that women ought to be warm, caring, and nurturing (Rudman and Glick, 2001; Heilman and Okimoto, 2007; Brescoll and Uhlmann, 2008; Gibson and Callister, 2010).

Hardly any research, however, has considered whether the benefits or penalties that men and women, respectively, face for expressing anger vary according to race (see Livingston et al., 2012 for an exception). This is notable because emerging research on the intersectionality of gender and race indicates that stereotypes and judgments of men and women vary considerably according to race (Johnson et al., 2012; Galinsky et al., 2013; Carpinella et al., 2015; Hall et al., 2015). For example, because behavioral norms differ for people of different genders and races, expressing anger could backfire for some groups. Consequently, the current study sought to replicate and extend past research on the relationship between gender, emotion expression, and status attainment at work by investigating whether advantages or penalties associated with emotion expression differ according to the race of the employee. Importantly, this intersectional perspective is not a hypothesis to be supported, but rather a framework from which to approach research on prejudice and stereotyping (Crenshaw, 1993). A positive finding (that a bias is different for targets of different races) and a null finding (that a bias generalizes in new contexts not previously studied) are both important contributions to our understanding.

In addition to gender, individuals may incur penalties (or rewards) for behavior depending upon whether such behavior conforms to the stereotypes of their race. Burgeoning research on intersectionality and multiple identities suggests that race and gender may interact and uniquely shape social judgment (Johnson et al., 2012; Galinsky et al., 2013; Carpinella et al., 2015; Hall et al., 2015; Kang and Bodenhausen, 2015). Early work found that the gender categorization of faces of Black and White men occurred more quickly than categorization of women’s faces, which suggested that people implicitly view the prototypical Black or White person as male (Zarate and Smith, 1990). Furthermore, other work suggests although some racial stereotypes apply equally to men and women, other stereotypes are more closely associated with a single gender—most often men (Eagly and Kite, 1987). Of particular relevance to the current investigation, Ghavami and Peplau (2013) found that Latina women were not regarded as aggressive, but Latino men were. White women were regarded as emotional and ditsy, whereas White men were regarded as assertive and leaders. Although the generalizability of these findings remains unclear, they nevertheless demonstrate how race and gender can interact in ways that shape expectations for behavior. This suggests that reactions to individuals that conform to or violate cultural stereotypes may vary depending on whether the target is male or female.

Indeed, people perceive certain races themselves to be gendered. Being Black is associated with masculinity while being Asian is associated with femininity, relative to being White (Johnson et al., 2012; Galinsky et al., 2013; Hall et al., 2015). One of the first studies to examine this phenomenon found that this effect is driven by shared stereotypes among the categories “Black” and “men” and “Asian” and “women,” as well as shared facial phenotypes among the categories “Black” and “men” (Johnson et al., 2012). Consistent with these findings, other work has found that Black candidates are more likely and Asian candidates are less likely to be selected for masculine leadership positions relative to White candidates (Galinsky et al., 2013). Of course, peoples’ identities are dynamic and multi-faceted. Nevertheless, identities that intersect across groups (e.g., Black and woman) may activate seemingly contradictory group-based stereotypes (e.g., Black and feminine) among perceivers. Past theory suggests that when this occurs, people’s minds may automatically activate one category and inhibit the other (Macrae et al., 1995). Such activation might come from viewing stereotype consistent behavior. For instance, a Black man playing basketball (race consistent) would lead a perceiver’s mind to activate the racial category Black and inhibit the category man. A Black man chopping wood (gender consistent) would activate the category man while Black would be inhibited (Macrae et al., 1995). However, when and why gender relative to race may be perceived as the more salient or relevant category for social judgment is still not well-understood. Moreover, the extent to which such nuanced stereotypes predict status perceptions differently for men and women of different races remains unclear. To our knowledge, no previous study has investigated whether men and women of different races are accorded status differentially according to their emotional expression. However, in an investigation of a similar question, researchers examined whether White and Black targets were perceived differently by gender for expressing dominance as a leader (Livingston et al., 2012). They found that White women and Black men who demonstrated a dominant leadership style were evaluated as having lower status than dominant White men and Black women. In other words, demonstrating dominance in leadership was penalized in White women and Black men but viewed as acceptable for White men and Black women. The researchers reasoned that Black women were spared negative evaluations in this context because they occupied dual identities (i.e., Black and female) with conflicting descriptive (e.g., “Black people are aggressive”) and prescriptive stereotypes (e.g., “women ought to be communal”), which buffered them from the perception of having clearly violated expectations. Therefore, Black women might not be penalized as harshly as White women or Asian women for aggressive behavior (Livingston et al., 2012; Ridgeway and Kricheli-Katz, 2013). Such prior work, however, has not examined how emotion expression from people with intersecting identities influences status perceptions in the workplace. Thus, the current study aimed to address this gap in the literature.

The current study extended previous research by assessing how both race and gender jointly influence status and competence evaluations in the workplace. Competence is closely related to status, but the constructs are distinct. People respect those who are competent; thus, competence is often viewed as both an antecedent and consequence of having high status (Tiedens, 2001; Fiske et al., 2002; Brescoll and Uhlmann, 2008). Consistent with previous findings, we hypothesized that relative to sadness, displays of anger would be rewarded with higher competence and status evaluations, and that men would be evaluated as more competent and awarded greater status than women. Further, we predicted a two-way interaction between gender and emotion such that angry women would be especially disadvantaged in status ratings. This finding was expected given previous findings that angry women were conferred lower status relative to angry men and sad women. Finally, because little research in this area has investigated the influence of race on these patterns, analyses of the influence of race were exploratory.

## Materials and Methods

### Participants

Students at a large university in southern California (*N* = 930) participated in an online experiment for partial credit in psychology courses. Data were omitted from 32% of initial participants; 100 participants who failed a directed query attention check instructing participants to give a specific response (Abbey and Meloy, 2017), and 200 participants who failed to accurately identify the target’s race or gender. Recent research recommends including attention checks for online data collection and excluding inattentive participants from analyses (Fleischer et al., 2015). Although, exclusion rates are expected to increase when studies use more than one attention check, our exclusion percentage falls within the normal range (i.e., 1.3–39.5%) (Abbey and Meloy, 2017). The final sample included 630 participants who were mostly young (*M*_*age*_ = 20.88, *SD* = 4.24; 4% unknown), racially diverse (39% Asian/Pacific Islander, 32% Latino/a/x, 14% White, 2% Black, 4% Middle Eastern, 5% other, and 4% unknown), and female (76%, 19% men, 1% other, and 4% unknown). This study was approved by the UC Irvine IRB, HS#2014-1430. Participants completed the study entirely online. Before beginning the study protocol, they were shown a description of the study procedures and informed that their participation was entirely voluntary.

### Design and Procedure

Participants completed an online study in which they assumed the role of a human resource manager at a consulting firm and were tasked with rating a potential employee. To keep participants naïve to our hypotheses, they were informed that human resource managers in large companies routinely have to make quick decisions about new employees concerning compensation and that the goal of this study was to determine how useful brief employee summaries are for determining new employees’ places within a company. Participants were then shown a written vignette describing a job applicant. Vignettes differed across participants in a between-subject design with respect to the applicant’s gender (male vs. female), race (Black, White, Asian, or Latino/a/x), and emotional response to being challenged by a coworker (anger vs. sadness). These three variables were fully crossed, yielding 16 conditions. The dependent variables were ratings of the applicant’s competence and accorded status.

In line with previous research, including experiments and field studies, we used subtle psychological cues to indicate applicants’ race and gender (Bertrand and Mullainathan, 2004; King et al., 2006; DeSante, 2013; Galinsky et al., 2013; Milkman et al., 2015; Butler and Homola, 2017; Gaddis, 2017a, b; Quillian et al., 2017). Such research has demonstrated that simply changing targets’ names (e.g., Emily and Greg vs. Lakisha and Jamal) alters their perceived race and gender, which subsequently leads to differential evaluations on outcomes like job application callbacks, government assistance awards, and ratings of applicant suitability (Bertrand and Mullainathan, 2004; King et al., 2006; DeSante, 2013; Galinsky et al., 2013; Milkman et al., 2015; Butler and Homola, 2017; Gaddis, 2017a, b; Quillian et al., 2017). Although some previous work has used photographs or video clips to manipulate a target’s perceived gender or race (e.g., Tiedens, 2001; Brescoll and Uhlmann, 2008; Livingston et al., 2012), a recent meta-analysis found that manipulating race via racially distinct names or visual cues yielded similar results (Quillian et al., 2017). Thus, we chose to subtly manipulate race and gender to appear more consistent with the type of information to which a human resource manager would have access (i.e., text description vs. photograph). Moreover, manipulating target names instead of group affiliations listed on a résumé (e.g., Black Student Union or Women in Business) has been shown to be a common and reliable method of indicating a target’s race and gender. Furthermore, this method limits the risk of inadvertently conveying additional information about a target’s potential attitudes, which could influence results (Bertrand and Mullainathan, 2004).

As such, participants were shown one of 16 brief vignettes, adapted from previous research (Judge et al., 2012), describing an applicant and told that some of the information was gathered from the applicant’s letters of recommendation and interviews. We manipulated both race and gender in two ways. To manipulate gender, in addition to using gender distinct names, we also listed the applicant’s sex (i.e., “male” or “female”) and used gender specific pronouns (i.e., “he” or “she”) in the vignette. Consistent with previous research (Bertrand and Mullainathan, 2004; King et al., 2006; DeSante, 2013; Galinsky et al., 2013; Milkman et al., 2015; Butler and Homola, 2017; Gaddis, 2017a, b), we manipulated race using racially distinct names (White: Gregory Clark and Jessica Miller; Black: DeShawn Thomas and Latoya Moore; Asian: Andrew Huang and Jennifer Chen; Latino/a/x: Chris Martinez and Anna Hernandez). The first names used for Black and White targets were taken from previous research (Bertrand and Mullainathan, 2004; Gaddis, 2017a). However, because fewer studies have investigated Latino/a/x and Asian American names, we used common surnames to indicate racial distinctiveness (Lauderdale and Kestenbaum, 2000; Comenetz, 2016; Gaddis, 2017b). Specifically, Chen and Huang are among the top 10 most common Chinese American names (Lauderdale and Kestenbaum, 2000) and Hernandez and Martinez are among the top five most common Latino/a/x names in the United States (Comenetz, 2016). Additionally, we explicitly stated the employee’s race in the application materials to eliminate any ambiguity and ensure that our stimuli sufficiently signaled the target’s race (Galinsky et al., 2013; Gaddis, 2017a). We also standardized each applicant’s education history to reduce judgments of socioeconomic status (see Butler and Homola, 2017; Gaddis, 2017a). For example, one vignette provided the following information:

Name: Gregory Clark. Applying for: Marketing and Sales Associate. Gender: Male. Ethnicity: White/European-American. Degree: B.A., Business, Stanford University. Interview Summary from Human Resources representative: He was well organized. His non-verbal behaviors were appropriate. Demonstrated great intelligence via college transcripts. Has good insights on topics. Observation: he became angry when challenged by a coworker.

The content of the other 15 vignettes was identical except for the employee’s name, gender, race, and whether the employee became “angry” or “sad” when challenged by a coworker.

Using a measure adapted from previous research (Brescoll and Uhlmann, 2008), participants evaluated the applicant on scales ranging from 1 (*none*) to 11 (*a great deal*) with respect to how much status, power, and independence the applicant deserved in their job. Participants were also asked to suggest a starting salary for their applicant and were told that the average compensation for the position was between $35,500 and $48,000 (Judge et al., 2012). We combined participants’ ratings of status, power, independence, and salary to create a composite measure of status, which is in line with how other scholars conceptualize status (Kessler-Harris, 2014).

To evaluate the applicant’s competence, we adapted a measure from previous research (Judge et al., 2012) and asked participants to evaluate the statements “the applicant was capable” and “the applicant was competent” on 5-point scales (1 = *strongly disagree*; 5 = *strongly agree*).

After completing a brief filler task, participants then recalled the applicants’ race and gender. We measured how much participants remembered about the applicant they evaluated, by asking, “What was the gender of your applicant,” (response options: “Male,” “Female,” or “I don’t remember”) and then “What was the race of your applicant” (response options: “White,” “Black,” “Asian,” “Latino,” or “I don’t remember”). Because this information was stated explicitly in the vignette, this served as a manipulation check. Finally, participants completed a demographic survey and were fully debriefed about the true nature of the experiment.

### Measures

#### Competence

A single measure of competence was computed by averaging participants’ evaluations of how capable and competent targets were. This measure exhibited acceptable reliability (*Cronbach’s α* = 0.75).

#### Status

To create the composite measure of status from participant’s ratings of status, power, independence, and salary, we first eliminated outlier salary responses greater than two standard deviations from the mean. Initial responses ranged from $20,000 to $60,000, responses without outliers ranged from $33,000 to $46,500. Next, we standardized participants’ responses to the four items by creating *Z*-scores and then computed the mean of the items. This measure exhibited acceptable reliability (*Cronbach’s α* = 0.75). Furthermore, confirmatory factory analyses showed that ratings of status, power, independence, and salary were better fit by a single-factor model, *X*^2^(2) = 5.31, *p* = 0.07, CFI = 1.00, TLI = 0.99, RMSEA = 0.05 (90% CI [0.00, 0.11], SRMR = 0.02, relative to a two-factor model, *X*^2^(1) = 4.02, *p* = 0.045, CFI = 1.00, TLI = 0.97, RMSEA = 0.07 (90% CI [0.01, 0.15], SRMR = 0.01. Therefore, we retained the single-factor model for our analyses.

## Results

### Preliminary Analyses

Because 300 participants were excluded from the sample for failing attention checks, we conducted two independent samples t-tests to examine whether the retained sample differed from excluded participants on our dependent variables. Results revealed that excluded participants (*M* = −0.07, *SD* = 0.71) compared to retained participants (*M* = 0.01, *SD* = 0.75) did not differ on status accorded to targets, *t*(840) = −1.41, *p* = 0.16. However, excluded participants (*M* = 3.90, *SD* = 0.72) relative to those included (*M* = 4.13, *SD* = 0.63) accorded targets lower competence ratings (adjusted for unequal variance), *t*(528.78) = −4.67, *p* < 0.001, *Cohen’s d* = −0.34, 95% CI [−0.48, −0.20]. Thus, we present analyses using participants who passed attention checks (*N* = 630) in the main document but include analyses using the full sample in Supplementary Material. Power analyses suggest sample sizes of 539 and 387 are needed to detect a small effect (*f* = 0.10) for an analysis of variance (ANOVA) model with a three-way interaction and 16 groups and an ANOVA model with a two-way interaction and 4 groups, respectively (Faul et al., 2007). As such, our primary analyses include a large enough sample sizes to be sufficiently powered.

Bivariate correlations among primary study variables, sample means, and means by condition are shown in Tables 1, 2. Target gender was positively associated with standardized status scores, such that an employee being a man was associated with being ascribed higher status. Interestingly, contrary to expectations, expressing anger was associated with being accorded less status. However, expressing anger was positively associated with competence ratings. Generally, target race and participant gender were not significantly associated with status conferral or competence ratings and thus were excluded. However, there was a positive association between single-item competence scores and participants being men (*r* = 0.08, *p* = 0.045) and a positive association between an employee being Black and standardized status scores (*r* = 0.12, *p* = 0.004) and the salary (*r* = 0.10, *p* = 0.02), status (*r* = 0.13, *p* = 0.001), and power (*r* = 0.09, *p* = 0.02) sub-components of composite status scores.

**TABLE 1:** Correlations and descriptive statistics among primary study variables.

	1	2	3	4	5	6	7	8	9	10
(1) Target gender (1 = men)	−									
	(630)									
(2) Target emotion (1 = angry)	0.00	−								
	(630)	(630)								
(3) Status (composite)^a^	0.10*	−0.15***	−							
	(581)	(581)	(581)							
(4) Salary	0.10*	−0.12**	0.59***	−						
	(584)	(584)	(581)	(584)						
(5) Status^b^	0.04	−0.14***	0.84***	0.28***	−					
	(629)	(629)	(581)	(583)	(629)					
(6) Power^b^	0.08*	−0.14***	0.81***	0.22***	0.71***	−				
	(629)	(629)	(581)	(583)	(628)	(629)				
(7) Independence^b^	0.08*	–0.06	0.77***	0.24***	0.54***	0.54***	−			
	(628)	(628)	(581)	(582)	(627)	(628)	(628)			
(8) Competence (composite)^c,d^	0.02	0.20***	0.31***	0.20***	0.26***	0.21***	0.22***	−		
	(630)	(630)	(581)	(584)	(629)	(629)	(628)	(630)		
(9) Capable^d^	0.03	0.17***	0.26***	0.16***	0.22***	0.18***	0.18***	0.89***	−	
	(630)	(630)	(581)	(584)	(629)	(629)	(628)	(630)	(630)	
(10) Competent^d^	0.00	0.18***	0.30***	0.19***	0.24**	0.20***	0.21***	0.90***	0.60***	−
	(630)	(630)	(581)	(584)	(629)	(629)	(628)	(630)	(630)	(630)
Mean			0.00	$39,617	7.19	6.59	7.38	4.13	4.16	4.09
Standard deviation			1.00	$2,920	1.81	1.96	1.88	0.64	0.69	0.73
*n*			(581)	(584)	(629)	(629)	(628)	(630)	(630)	(630)

n is listed in parentheses beneath each correlation. Target race is not included because no significant effects with status or competence emerged. ^*a*^Standardized composite of salary, status, power, and independence variables. ^*b*^Variable ranges from 1 to 11. Higher numbers indicate increases. ^*c*^Composite of capable and competent variables. ^*d*^Variable ranges from 1 to 5. Higher number indicate increases. *p < 0.05, **p < 0.01, ***p < 0.001.

**TABLE 2:** Means by condition for status and competence variables.

	Competence^a^											
Group	White			Black			Asian			Latino/a/x		
	*n*	*M*	*SD*	*n*	*M*	*SD*	*n*	*M*	*SD*	*n*	*M*	*SD*
Angry women	37	4.11	0.49	41	4.34	0.54	39	4.14	0.54	45	4.22	0.47
Angry men	41	4.22	0.57	37	4.11	0.49	39	4.24	0.60	37	4.27	0.66
Sad women	36	4.00	0.72	35	3.91	0.79	45	3.93	0.80	39	4.22	0.58
Sad men	34	3.93	0.51	36	4.00	0.61	39	4.05	0.57	41	4.00	0.63

	**Status^b^**											
**Group**	**White**			**Black**			**Asian**			**Latino/a/x**		
	***n***	***M***	***SD***	***n***	***M***	***SD***	***n***	***M***	***SD***	***n***	***M***	***SD***

Angry women	33	–0.41	0.88	37	–0.11	0.67	36	–0.33	0.78	44	–0.15	0.93
Angry men	40	–0.20	0.71	39	0.41	0.71	36	–0.06	0.68	35	0.02	0.80
Sad women	32	0.09	0.61	31	0.10	0.63	43	0.05	0.80	38	0.21	0.78
Sad men	33	0.24	0.67	34	0.24	0.58	33	0.04	0.51	37	0.01	0.65

**^*a*^Composite of capable and competent variables (ranges from 1 to 5). ^*b*^Standardized composite of salary, status, power, and independence variables*.*

### Primary Analyses

To test the predicted effects of a target gender and emotional expression and the exploratory impact of target race on evaluations of employees’ competence, we conducted a 4 (applicant race: White, Black, Asian, and Latino/a/x) by 2 (applicant gender: women, men) by 2 (emotion: sad, angry) ANOVA (see Table 3). Results revealed a main effect of emotion. As expected, participants evaluated applicants as more competent if they expressed anger in response to being challenged (adjusted *M* = 4.24, *SE* = 0.04) than if they expressed sadness (adjusted *M* = 4.00, *SE* = 0.04). contrary to our prediction, no significant effects were observed for employee gender. Additionally, there were no effects of target race or any of the interactions. For each primary analysis reported in the manuscript, we also conducted supplementary analyses that included participants who failed attention checks, as well as alternative analyses accounting for potential covariates. Generally, supplementary analyses produced patterns consistent with those reported in the results (see Supplementary Material for details).

**TABLE 3:** Three-way ANOVA models for competence and standardized status.

Variable	Competence^a^ (*n* = 630)					
	Partial SS	*df*	*F*	*p*	η^2^/$\eta_{\text{p}}^{2}$	95% CI
Model	15.22	15	2.63	< 0.001	0.06	[0.01, 0.08]
Race	1.22	3	1.05	0.370	0.01	[0.00, 0.02]
Gender	0.06	1	0.15	0.703	0.00	[0.00, 0.01]
Emotion	9.58	1	24.79	< 0.001	0.04	[0.01, 0.07]
Race × gender	0.78	3	0.67	0.570	0.00	[0.00, 0.01]
Race × emotion	2.30	3	1.98	0.115	0.01	[0.00, 0.03]
Gender × emotion	0.58	1	1.49	0.223	0.00	[0.00, 0.02]
Race × gender × emotion	0.45	3	0.42	0.739	0.00	[0.00, 0.01]
Residual	237.25	614				
Total	252.47	629				

**Variable**	**Status^b^ (*n* = 581)**					
	***Partial SS***	***df***	***F***	***p***	**η^2^/$\eta_{\text{p}}^{2}$**	**95% CI**

Model	25.12	15	3.18	< 0.001	0.08	[0.02, 0.10]
Race	5.04	3	3.20	0.023	0.02	[0.00, 0.04]
Gender	3.45	1	6.56	0.011	0.01	[0.00, 0.04]
Emotion	7.20	1	13.70	< 0.001	0.02	[0.01, 0.05]
Race × gender	2.18	3	1.38	0.248	0.01	[0.00, 0.02]
Race × emotion	3.61	3	2.29	0.078	0.01	[0.00, 0.03]
Emotion × gender	2.71	1	5.16	0.024	0.01	[0.00, 0.03]
Race × emotion × gender	0.56	3	0.36	0.784	0.00	[0.00, 0.01]
Residual	297.15	565				
Total	322.26	580				

**Eta-squared values for individual model terms are partial. ^*a*^Composite of capable and competent variables (ranges from 1 to 5). ^*b*^Standardized composite of salary, status, power, and independence variables*.*

**FIGURE 1:**
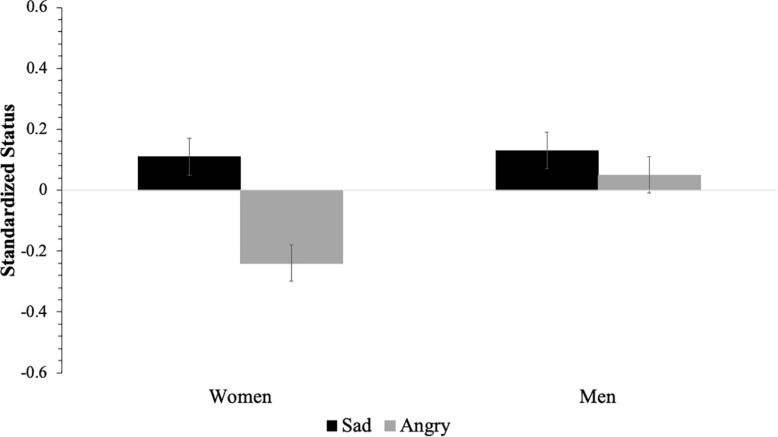
Interaction between applicant gender and emotion. Error bars represent ± one standard error.

To test our *a priori* hypothesis that expressing anger would be detrimental for the workplace status of women but not men, we conducted a 2 (applicant gender: women and men) by 2 (emotion: sad and angry) ANOVA on standardized composite status scores. Counter to our hypothesis, results revealed a significant main effect for emotion, *F*(1,577) = 12.73, *p* < 0.001, $\eta_{\text{p}}^{2}$ = 0.02, 95% CI [0.00, 0.05], such that angry employees (adjusted *M* = *−*0.10, *SE* = 0.04) were accorded less status than sad employees (adjusted *M* = 0.12, *SE* = 0.04). In line with our prediction, we also found a significant main effect of gender, *F*(1,577) = 6.05, *p* = 0.01, $\eta_{\text{p}}^{2}$ = 0.01, 95% CI [0.00, 0.03], such that employees who were men (adjusted *M* = 0.09, *SE* = 0.04) were accorded more status than women (adjusted *M* = −0.07, *SE* = 0.04). These effects were qualified by a significant interaction between gender and emotion, which is displayed in Figure 1, *F*(1,577) = 4.87, *p* = 0.03, $\eta_{\text{p}}^{2}$ = 0.01, 95% CI [0.00, 0.03]. Bonferroni adjusted pairwise comparisons revealed that sad men (adjusted *M* = 0.13, *SE* = 0.06) and women (adjusted *M* = 0.11, *SE* = 0.06) were not accorded different levels of status, *t*(575) = 0.18, *p* = 0.86, and neither were sad and angry men [adjusted *M* = 0.05, *SE* = 0.06; *t*(575) = −0.96, *p* = 0.34]. However, angry women (adjusted *M* = −0.24, *SE* = 0.06) were accorded lower status than sad women, *t*(579) = 4.11, *p* < 0.001, 95% CI [−0.52, −0.18], sad men, *t*(579) = 4.24, *p* < 0.001, 95% CI [−0.54, −0.20], and angry men, *t*(579) = 3.36, *p* = 0.001, 95% CI [0.12, 0.45].

We then conducted an exploratory analysis to test whether evaluations of employees’ status differed based on a target’s race, gender, and emotion by conducting a 4 × 2 × 2 ANOVA (see Table 3). Results revealed significant main effects of race, gender, and emotion. Overall, women (adjusted *M* = −0.07, *SE* = 0.04) were accorded less status than men (adjusted *M* = 0.09, *SE* = 0.04), and people who expressed anger (adjusted *M* = −0.10, *SE* = 0.04) were accorded less status than people who expressed sadness (adjusted *M* = 0.13, *SE* = 0.04). Bonferroni corrected pairwise comparisons between applicants’ race revealed that Black applicants (adjusted *M* = 0.16, *SE* = 0.06) were accorded more status than White (adjusted *M* = −0.08, *SE* = 0.06), *t*(579) = 2.70, *p* = 0.007, 95% CI [0.06, 0.41] and Asian (adjusted *M* = −0.08, *SE* = 0.06), *t*(579) = −2.77, *p* = 0.006, 95% CI [−0.41, −0.07] applicants. The main effects were qualified by an interaction between gender and emotion, which was consistent with the pattern displayed in Figure 1. No significant effects were found for any other interaction.

### *Post hoc* Analyses

Given the racial diversity of our sample, we conducted additional exploratory analyses to explore whether the patterns for gender and emotion on status differed among Asian, Latino/a/x, and White participants. As such, we conducted a 3 (participant race: Asian, Latino/a/x, and White) by 2 (gender) by 2 (emotion) ANOVA (see Table 4). We did not examine Black participants separately because they only comprised 2% of our sample. Results revealed a main effect of emotion that was qualified by an interaction between gender and emotion, which was again consistent with the pattern in Figure 1. The three-way interaction was not significant, suggesting that the gender by emotion interaction pattern does not differ by racial group. It is important to note that our sample size was sufficient to detect two-way interactions but not a three-way interaction; thus, these exploratory results should be interpreted with caution.

**TABLE 4:** Effects of gender, emotion, stereotype consistency and participant race on standardized status.

Variable	Status^a^ (*n* = 514)					
	Partial SS	df	*F*	*p*	η^2^/$\eta_{\text{p}}^{2}$	95% CI
Model	14.86	11	2.42	0.006	0.06	[0.01, 0.07]
Participant race (prace)	0.30	2	0.27	0.762	0.00	[0.00, 0.01]
Gender	1.42	1	2.53	0.112	0.01	[0.00, 0.02]
Emotion	6.31	1	11.28	< 0.001	0.02	[0.00, 0.05]
Prace × gender	0.93	2	0.83	0.435	0.00	[0.00, 0.02]
Prace × emotion	0.27	2	0.24	0.784	0.00	[0.00, 0.01]
Gender × emotion	3.27	1	5.85	0.016	0.01	[0.00, 0.04]
Prace × gender × emotion	0.64	2	0.57	0.567	0.00	[0.00, 0.01]
Residual	280.70	502				
Total	295.56	513				

**Variable**	**Status^a^ (*n* = 514)**					
	**Partial SS**	**df**	***F***	***p***	**η^2^/$\eta_{\text{p}}^{2}$**	**95% CI**

Model	14.86	11	2.42	0.006	0.06	[0.01, 0.07]
Prace	0.30	2	0.27	0.762	0.00	[0.00, 0.01]
Gender	1.42	1	2.53	0.112	0.01	[0.00, 0.02]
Stereotype consistency (SC)	3.27	1	5.85	0.016	0.01	[0.00, 0.04]
Prace × gender	0.93	2	0.83	0.435	0.00	[0.00, 0.02]
Prace × SC	0.64	2	0.57	0.567	0.00	[0.00, 0.01]
SC × gender	6.31	1	11.28	< 0.001	0.02	[0.00, 0.05]
Prace × SC × gender	0.27	2	0.24	0.784	0.00	[0.00, 0.01]
Residual	280.70	502				
Total	295.56	513				

**Eta-squared values for individual model terms are partial. ^*a*^Standardized composite of salary, status, power, and independence variables*.*

**FIGURE 2:**
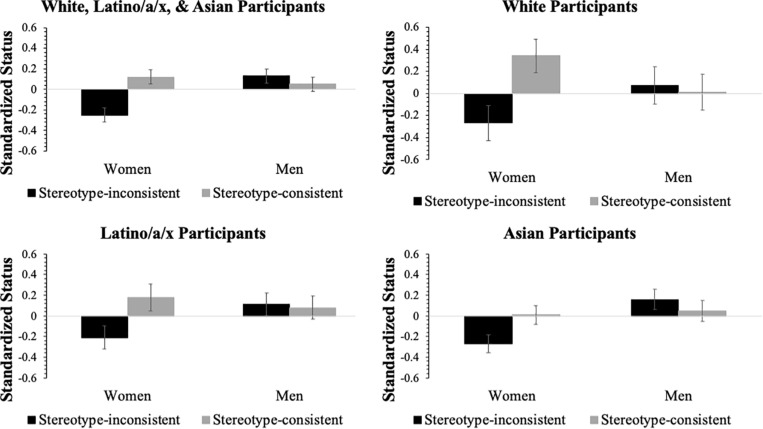
Interactions between applicant gender and stereotype consistency by participant race. Error bars represent ± one standard error.

To further probe the relationship between status, applicant gender, and expressed emotion we recategorized our data to explore an additional factor—stereotype consistency of gendered emotion expression. As research demonstrates, expressing certain emotions is socially sanctioned depending on a person’s gender (Brescoll, 2016). Men are permitted to express emotion associated with dominance (i.e., anger) and women are permitted to express emotions that convey vulnerability (e.g., sadness). To test whether stereotype consistency and gender influenced status conferral, we created a dummy coded stereotype consistency variable where angry men and sad women (stereotype-consistent) were coded 1 and sad men and angry women (stereotype-inconsistent) were coded 0. We then conducted a 3 (participant race) by 2 (stereotype-consistency: consistent, inconsistent) by 2 (gender) ANOVA (see Table 4). We found a significant main effect of stereotype consistency qualified by a two-way interaction between gender and stereotype consistency (see Figure 2). Bonferroni corrected pairwise comparisons revealed that stereotype-inconsistent women (adjusted *M* = −0.25, *SE* = 0.07) were accorded lower status than stereotype-consistent women (adjusted *M* = 0.12, *SE* = 0.07), *t*(512) = 4.00, *p* < 0.001, 95% CI [0.19, 0.56]. There was no difference between stereotype-consistent men (adjusted *M* = 0.05, *SE* = 0.07) and stereotype-inconsistent men (adjusted *M* = 0.13, *SE* = 0.07), *t*(512) = −0.81, *p* = 0.42. However, stereotype-inconsistent women were accorded lower status than stereotype-inconsistent men, *t*(512) = 4.04, *p* < 0.001, 95% CI [0.19, 0.56] and stereotype-consistent men, *t*(512) = 3.23, *p* = 0.001, 95% CI [0.12, 0.48].

Although the three-way interaction was not significant, we further explored the gender by stereotype consistency interaction among White, Latino/a/x, and Asian participants in three separate Bonferroni corrected ANOVA models and examined potential pairwise differences (see Figure 2). For White participants, there were no significant main effects for gender, *F*(1,79) = 0.00, *p* = 0.97, or stereotype consistency, *F*(1,79) = 2.94, *p* = 0.09. The interaction reached traditional levels of significance but did not pass the threshold for the Bonferroni adjusted levels, *F*(1,79) = 4.28, *p* = 0.04, $\eta_{\text{p}}^{2}$ = 0.06, 95% CI [0.00, 0.17]. Nevertheless, we proceeded with Bonferroni corrected pairwise comparisons and found that stereotype-inconsistent women (adjusted *M* = −0.27, *SE* = 0.16) were accorded lower status that stereotype-consistent women (adjusted *M* = 0.33, *SE* = 0.15), *t*(81) = 2.79, *p* = 0.007, 95% CI [0.17, 1.03]. Among Latino/a/x participants, neither the main effects for gender, *F*(1,188) = 0.93, *p* = 0.34 and stereotype consistency, *F*(1,188) = 2.46, *p* = 0.12, nor the interaction, *F*(1,188) = 3.39, *p* = 0.07, reached traditional levels of significance. Pairwise comparisons revealed stereotype-inconsistent women (adjusted *M* = −0.21, *SE* = 0.11) were accorded lower status than stereotype-consistent women (adjusted *M* = 0.18, *SE* = 0.13), *t*(189) = 2.32, *p* = 0.021, 95% CI [0.06, 0.72] and stereotype-inconsistent men (adjusted *M* = 0.11, *SE* = 0.11), *t*(189) = 2.04, *p* < 0.043, 95% CI [0.10, 0.63]; although, neither comparison met the Bonferroni corrected level of significance. For Asian participants, there was no main effect for stereotype consistency, *F*(1,235) = 0.74, *p* = 0.39. There was a significant Bonferroni corrected main effect for gender, *F*(1,235) = 6.21, *p* = 0.013, $\eta_{\text{p}}^{2}$ = 0.03, 95% CI [0.00, 0.08], such that men (adjusted *M* = 0.11, *SE* = 0.07) were accorded more status than women (adjusted *M* = −0.13, *SE* = 0.07) but the interaction did not meet the adjusted level of significance, *F*(1,235) = 4.51, *p* = 0.035, $\eta_{\text{p}}^{2}$ = 0.02, 95% CI [0.00, 0.07]. Pairwise comparisons revealed stereotype-inconsistent women (adjusted *M* = −0.27, *SE* = 0.09) were accorded lower status than stereotype-consistent women (adjusted *M* = 0.01, *SE* = 0.09), *t*(237) = 2.17, *p* = 0.031, 95% CI [0.03, 0.54], stereotype-consistent men (adjusted *M* = 0.05, *SE* = 0.10), *t*(237) = 2.35, *p* = 0.019, 95% CI [0.05, 0.58], and stereotype-inconsistent men (adjusted *M* = 0.16, *SE* = 0.10), *t*(237) = 3.29, *p* = 0.001, 95% CI [0.17, 0.70]. However, only the comparison between stereotype-inconsistent women and stereotype-inconsistent men reached the adjusted significance level.

## Discussion

The present study investigated how people evaluate expressions of emotion in the workplace among employees of different races and genders. In accordance with prior research, participants rated employees who expressed anger as more competent than those who expressed sadness irrespective of race or gender. Although employees who expressed anger were perceived to be more competent than their sad counterparts, these assessments of competence did not lead participants to accord those employees higher status. In fact, counter to previous research, we found that participants accorded less status to employees who expressed anger relative to sadness. In line with past findings, however, we did find some evidence that stereotype violations of gendered emotion expression (i.e., angry women) were associated with women being accorded low status. Overall, women who expressed anger (i.e., stereotype-inconsistent) were accorded the least amount of status relative to women who expressed sadness (i.e., stereotype-consistent) and men who expressed either anger (i.e., stereotype-consistent) or sadness (i.e., stereotype-inconsistent). Furthermore, and counter to previous findings (Tiedens, 2001; Brescoll and Uhlmann, 2008), there were no differences in status between men who expressed anger or sadness.

Our findings for women who expressed anger are in line with past research showing that when people violate prescriptive norms, their behavior is often perceived negatively (Heilman and Okimoto, 2007). Women who present themselves as self-confident and assertive are sometimes rated as highly competent and capable of leadership, but also as socially deficient and unlikable by both male and female perceivers (Rudman and Phelan, 2008). The present study extends these findings to show that, across races, women who violated gender norms by expressing anger were perceived as highly competent but were nonetheless recommended for lower status.

These results vary slightly from prior work, which has found that competence perceptions correlate very closely with status judgments (Tiedens, 2001; Brescoll and Uhlmann, 2008) and that women who express anger are viewed as less competent and less deserving of status relative to men who expressed anger or women who expressed sadness (Brescoll and Uhlmann, 2008). Competence and status are closely related but not synonymous. Social status is commonly defined as the degree to which you are respected and admired by others (Magee and Galinsky, 2008). People respect those who are competent; thus, competence is often viewed as both an antecedent and consequence of having high status (Tiedens, 2001; Fiske et al., 2002; Brescoll and Uhlmann, 2008). However, other factors (e.g., likeability) may influence whether or not perceptions of competence translate into admiration or accorded status. These results also refine our understanding of the relationship between emotion expression, competence, and status perceptions for men versus women. Although competence is generally thought to play a larger role than likeability in driving status perceptions (Tiedens, 2001), the fact that angry women were viewed as competent but awarded lower status suggests that perceived competence may be less of a driver for status perceptions for women at work (see Phelan et al., 2008). Other factors, including likeability, may play a relatively larger role for women than they do for men, which would explain this discrepancy between competence and status for women. Past research has documented a “double bind” in which women can be perceived as competent for acting strong and assertive, but they are usually disliked (Oakley, 2000). Our study may be capturing one way in which people seek to penalize women (relative to men) for expressing anger, even while they recognize that they demonstrate competence. Future research should further unpack this issue of the relative weight of competence versus likeability on status perceptions for men versus women.

Given the consistent pattern of women being penalized in the workplace for expressing anger future research should investigate ways to reduce such evaluative demerits given to women. Indeed, research has shown that attributing women’s anger to external rather than internal reasons (Brescoll and Uhlmann, 2008; Barrett and Bliss-Moreau, 2009) or demonstrating evidence of being communal (Heilman and Okimoto, 2007) mitigates the negative backlash women face for expressing anger or simply being successful. However, it is not incumbent upon those being harmed by stereotypes to rectify the disparate outcomes. Rather organizations should institute policies and practices to account for the evaluative bias women experience. For instance, because research has revealed bias against women and non-White faculty in student teaching evaluations (Chisadza et al., 2019) some universities take that bias into consideration and reduce the weight of student evaluations when assessing promotions for faculty whom would be affected by such bias. Another tactic that research suggests helps reduce evaluative bias is to inform evaluators of such biases (Peterson et al., 2019). In other words, alerting employees and managers that such biases might creep into their evaluations of women employees who occupy higher and lower occupational position might successfully reduce bias in evaluations of their accorded status. Despite our findings of angry women being penalized, research demonstrates that restraining anger expressions is commonly used by both men and women managers, which suggests that interpersonal anger expressions violate expected social norms in the workplace, and that neither men or women should seek to express anger (Domagalski and Steelman, 2007; Geddes et al., 2020). Nevertheless, because anger expression does happen in the workplace and, in response, women face disparate impacts relative to men, it is important for organizations to achieve parity in the consequences of such emotion expression.

In addition to our findings for angry women aligning with previous research, we also found surprising patterns for sad relative to angry men. In line with past findings (e.g., Brescoll and Uhlmann, 2008), we expected angry men to be accorded the most status. We found, however, these there was no difference in status for angry and sad men. One reason for this pattern could be that our sample was comprised of mostly women. Although we did observe an association suggesting male participants accorded higher competence ratings, given the small sample of men, the expected effects may have been blunted. Another reason for the observed patterns might be the racial make-up of our sample. Previous work has demonstrated that White Americans might have more differentiated expectations of gender stereotyped emotion expression (e.g., men express anger and women express sadness) relative to Black, Latino/a/x, and Asian Americans (Durik et al., 2006). That is, White Americans report higher expectations for White women to express sadness and White men to express anger compared to Latino/a/x, and Asian Americans (Durik et al., 2006). Thus, it may be that because our sample was predominately Asian and Latino/a/x American, the patterns evaluating stereotype-consistency were not as strong they have been with previous research using mostly White samples. Therefore, an important contribution of this study is evidence that participant race may influence the link between gender, emotional expression, and status conferral. Approximately 80% of our sample classified themselves as non-White. In contrast, much research in this domain, has either not reported participant race (e.g., Rudman and Glick, 2001; Tiedens, 2001; Brescoll and Uhlmann, 2008) or used primarily White participant samples (e.g., Judge et al., 2012). Although the current study intended to explore how race and gender may interact to influence perceptions of targets’ emotional expressions, we did not intentionally factor participant race into our design given that stereotype research tends not to find significant differences in susceptibility to stereotypes across race or gender (Simon and Hamilton, 1994; Heilman, 2012). However, recent work has called on researchers to recognize how participants’ identities are linked to social positions that may lead them to have different perceptions of the same stimuli (McCormick-Huhn et al., 2019). Such recognition will allow researchers to more comprehensively understand the dynamics of the phenomena they are investigating, and better identify the boundary conditions. Nevertheless, the current study used an intersectional frame, and our sample demographics reveal how participant racial identity—a facet of intersectionality that is often given less attention—should be an important consideration that is factored into future research.

Related to considering intersectionality in this area of research, hardly any studies investigating gender and dominance in the workplace include race as a factor (Shields, 2002). Therefore, another contribution of the current research is the ability to differentiate whether or not the relative status advantages or penalties for expressing negative emotion are awarded to men and women across races. By manipulating both target race and gender, our findings advance the emerging literature on social perception at the intersection between race and gender. Although one prior study suggested that men and women might be perceived differently for expressing negative emotions at work depending upon their race (Livingston et al., 2012), the current investigation did not find support for that hypothesis.

There are a few possible reasons for this result. As mentioned, our participant sample was mostly non-White. And, because most of the participants likely evaluated employees of color, some of whom shared their racial in-group, the negative racialized stereotypes that influence White Americans’ judgments might not have been activated for those participants. Another potential explanation, as some other research has argued, is that because Black women’s social group membership is at the intersection of race and gender, they might experience both advantages and disadvantages relative to White women. For instance, one study found that, unlike White women leaders, Black women leaders were not penalized for acting agentically and were evaluated similarly to White men leaders (Livingston et al., 2012). The authors argued that, because Black women do not represent solely the “Black” prototype or the “female” prototype, they may not be penalized to the same extent for behavior that is counter-stereotypical for their race or gender, a phenomenon known as “intersectional invisibility” (Crenshaw, 1993; Purdie-Vaughns and Eibach, 2008; Biernat and Sesko, 2013; Thomas et al., 2014). Thus, Black women may benefit relative to White women when they express dominance as a leadership style, but not when they express anger.

Additionally, one study found that although angry Black women were perceived as more aggressive and hostile than angry White women, both groups were viewed as equally competent (for discussion see McCormick-Huhn et al., 2016). In the current study, it is possible that all angry women were accorded less status because of specific stereotypes associated with their respective racial groups. For instance, displaying anger is consistent with the “angry Black woman” stereotype, but being angry violates the “warm and nice” stereotype for White women. Thus, Black women might be accorded less status for validating a negative stereotype, whereas White women might be accorded less status for violating a positive one. Furthermore, Latina women and Asian women might show similar stereotypical patterns. For instance, the “spicy/loud Latina” stereotype might operate similarly to the “angry” or “sassy” Black woman stereotype, and the “meek” and “deferential” Asian woman stereotype might resemble the “warm,” “nurturing” stereotype of White women (Rosette et al., 2016). Indeed, a final potential explanation for not finding race differences among our target evaluations is that expressions of anger in a job candidate, wherein an individual is in a position of low power and is seeking approval from an interviewer, may be perceived as particularly counter-normative for White women, who are expected to be “communal,” and stereotype confirming for Black women, who are expected to be “angry.” In both cases, violating or confirming a stereotype may lead to equal status penalties relative to “angry” men. Given that previous research has found that women of different races are perceived as similarly competent even when they have different stereotypes (see McCormick-Huhn et al., 2016), it is possible that our findings demonstrate that this pattern also holds when women of different racial groups are accorded status. Moreover, our findings are in line with recent research indicating that the link between violating or conforming to stereotypes and status conferral is complex and new theoretical models (e.g., intersectional theory) are needed to better track potential associations (e.g., Crenshaw, 1993; Brescoll, 2016; McCormick-Huhn et al., 2016). Taken together, our results lend tentative evidence suggesting that regardless of race, women who express anger are penalized relative to men who express anger. Future research should consider how assertive actions versus emotional expressions and confirming or violating stereotypes affect status perceptions differently for men and women of different races, as well as how the context in which such behaviors or emotions are expressed may moderate these effects.

The current study is among the first to address workplace anger across both gender and race, but it is not without limitations. Although the racial diversity of our sample allowed us to add another dimension to our analytic approach, many of our exploratory *post hoc* analyses may not have achieved sufficient statistical power to detect significant effects. Nevertheless, many of our analyses did, in fact, yield significant results even with Bonferroni corrected alpha levels. Specifically, we found angry women were accorded lower status than sad women among White American participants and angry women were accorded lower status than sad men among Asian American participants. Such patterns offer some assurance that the effect of women receiving negative evaluations when expressing anger is robust. However, given analyses within the White, Latino/a/x, and Asian American racial groups were performed using smaller than required sample sizes, we must urge caution when interpreting these results.

Another potential limitation is that participants evaluated a stellar candidate with an impressive academic background and strong interviewing skills. When participants evaluate outstanding employees, they may take into account the hardships faced by members of stigmatized racial groups and augment those employees’ accomplishments (Kelley, 1973; Baron et al., 2001). This explanation, while speculative, is consistent with past research demonstrating contexts that influence when and how people use stereotypes (O’Brien et al., 2008). Moreover, recent research indicates that perceptions of socioeconomic status can interact with race and gender in ways that bias judgments (Kang and Bodenhausen, 2015). Participants may have attributed high socioeconomic status to our employees due to their prestigious education (Butler and Homola, 2017; Gaddis, 2017b). This issue highlights an important direction for future research. Asking participants to evaluate employees of different races and gender with a range of qualifications would permit researchers to evaluate the possibility of augmentation effects for judgments of highly qualified employees or employees of higher socioeconomic status.

It is also possible that reading a written vignette listing the participants’ race was not a powerful enough stimulus to activate stereotypes and bias judgments. Past research shows that manipulating race through text is sufficient to elicit bias or discrimination (Bertrand and Mullainathan, 2004; King et al., 2006; Milkman et al., 2012, 2015; DeSante, 2013; Galinsky et al., 2013; Butler and Homola, 2017; Gaddis, 2017a, b; Quillian et al., 2017). However, stereotypes associated with race and gender may be more strongly activated through visual representations like photos or videos than through text. Instead of using job application information alone, a number of past studies on emotion and status perceptions have asked participants to evaluate photos or video clips of prospective candidates (Tiedens, 2001; Brescoll and Uhlmann, 2008; Livingston et al., 2012). However, participants retained in our analyses correctly identified the target’s race and gender later in the study, indicating that race and gender had been clearly encoded, which gave opportunity for associated stereotypes to be activated. Nevertheless, an important area for future research would be to use visual stimuli (e.g., photographs) to explore whether interactions between race and gender driving status judgments emerge under different presentation contexts, which would increase external validity.

Another potential limitation is that our participants were university students, who may not have the same perceptions, experiences, and biases as actual hiring managers. Future research also ought to examine whether full-time work experience or managerial experience affects how employee emotional expressions affect status and competence conferral.

Finally, while this study represents an important contribution to intersectional research on prejudice, examining the full breadth of the of race, gender, and emotion is a complex and multifaceted challenge. We investigated how evaluations of competence and status varied based on four potential races, two potential genders, and two potential emotions, but further research is needed in order to fully understand other categories of all three variables. Because we did not directly test how neutral emotions were perceived when expressed by different genders and races, we cannot say how sadness and anger compare to neutral emotional expressions within men and women. However, previous work has found that neutral and angry emotional expressions are associated with comparable levels of competence when evaluating men (Hareli et al., 2013). Moreover, women are accorded lower status when expressing anger relative to no emotion (Brescoll and Uhlmann, 2008). Nevertheless, the current study can only make relative comparisons between men and women who express sadness or anger.

## Conclusion

The present results replicate past findings that women who express anger receive status penalties relative to men who express anger and women who express sadness (Brescoll and Uhlmann, 2008; Livingston et al., 2012). With increasing interest in recent years on the importance of successful replications in the social sciences (Open Science Collaboration, 2015), the replication of this finding further underscores the robustness of this bias. What is more, it extends past findings to show that across races, even when competence is judged to be high, women are penalized for expressing anger in the workplace relative to men (Brescoll and Uhlmann, 2008). Both stereotype violation and behavioral attributions may contribute to these results. People tend to make dispositional attributions for women’s emotional expressions but situational attributions for men’s (Brescoll and Uhlmann, 2008; Barrett and Bliss-Moreau, 2009). If expression of emotion in the workplace is viewed as indicative of personality for women but circumstance for men, people may consider women’s negative emotions to be more threatening. Thus, our findings underscore a dilemma that women across races experience in the workplace: to be taken seriously, one must be assertive and competent. To be accorded higher status, however, women cannot violate the stereotype that they are communal and cooperative. The fact that this bias exists against women of diverse racial backgrounds who get angry, despite the existence of different stereotypes about being a woman in that culture, highlights the robustness of this finding and adds nuance to our understanding of the ways in which stereotypes become activated and shape social judgment.

## Data Availability Statement

The original contributions presented in the study are included in the article/Supplementary Material, further inquiries can be directed to the corresponding author.

## Ethics Statement

The studies involving human participants were reviewed and approved by University of California, Irvine Institutional Review Board. The patients/participants provided their written informed consent to participate in this study.

## Author Contributions

CM helped to develop study materials, conducted statistical analyses, and drafted and edited the manuscript. KC developed study materials, oversaw data collection, conducted statistical analyses, and drafted and edited the manuscript. EF drafted and edited the manuscript. LL provided consultation for statistical analyses and drafted and edited the manuscript. All authors contributed to the article and approved the submitted version.

## Conflict of Interest

The authors declare that the research was conducted in the absence of any commercial or financial relationships that could be construed as a potential conflict of interest.
