# Supplementary material for: Workplace Anger Costs Women Irrespective of Race
Source: Front Psychol. 2020 Nov 6;11:579884. doi: 10.3389/fpsyg.2020.579884 (PMC7677348; doi:10.3389/fpsyg.2020.579884)
Supplement: Supplementary file 1 [file Table_1.pdf]

Supplementary Materials

Method

Participants

The base sample was comprised of 930 students at a large university in southern California. Out of the 930, 100 participants failed a directed query attention check instructing participants to give a specific response (Abbey and Meloy, 2017), and 200 participants failed to accurately identify the target's race or gender. The full sample was made up of mostly young (M_{age} = 20.84, *SD* = 3.95; 4% unknown), racially diverse (44% Asian/Pacific Islander, 29% Latino/a/x, 13% White, 2% Black, 4% Middle Eastern, 4% other, 4% unknown), women (74.5%, 21% men, .5% other, 4% unknown). The analyses reported herein were conducted using the full sample.

Potential Covariates

To account for participants' feelings toward different racial groups, participants completed four feeling thermometers assessing how favorable (0 = *unfavorable*; 100 = *favorable*) they perceived White, Black, Asian, and Latino racial groups. We averaged participants' ratings for Black, Asian, and Latino people and created a single "people of color" score (Cronbach's α = .78). Participants also completed the Motivation to Control Prejudice Reactions scale (Dunton and Fazio, 1997) where they used a 7-point Likert scale (1 = *strongly disagree*; 7 = *strongly agree*) to rate the extent to which they agreed with statements like, "it's important to me that other people not think I'm prejudiced" (Cronbach's α = .73). We also assed participants' self-reported gender and race.

Results

Preliminary Analyses

Bivariate correlations among all study variables and status and competence means by condition are shown in Supplementary Tables 1 and 2. Target gender was positively associated with status, such that an employee being a man was associated with being ascribed higher status. Contrary to expectations, expressing anger was associated with being accorded less status, but more competence. Participant gender was positively associated with composite status scores and independence ratings, such that participants who were men accorded higher status ratings. Positive feelings toward people of color was associated with according higher status and competence, and positive feelings toward White people was associated with according higher salary. Target race was also associated with status ratings. Being a White employee was associated with lower status scores and being a Black employee was associated with more status. Additionally, higher motivation to control prejudice was associated with evaluating targets as more competent. Participant race was also associated status and competence. Being a White or Latino/a/x American participant was associated with higher salaries and competence scores, whereas being an Asian American participant was associated with according less status and competence scores. Being a Latino/a/x American participant was associated with positive feelings toward people of color. Participants being men was associated with lower motivation to control prejudice, but increased motivation to control prejudice was associated with positive feelings for White people and people of color. Being a White American participant was

positively associated with positive feelings toward White people, being a Latino/a/x American participant was positively associated with positive feelings toward people of color, and being an Asian American participant was negatively associated with feelings toward White people and people of color.

Given the correlations between participant race, feelings toward White people and people of color, and motivation to control prejudice, we conducted a one-way MANOVA to examine potential mean differences between White, Asian, and Latino/a/x American participants (n = 796) on each measure. Results revealed a significant main effect of participant race, Wilk's λ = .87, $F(1, 778) = 18.16$, $p < .001$, $\eta_p^2 = .05$, 95% CI [0.02, 0.08]. Probing the individual ANOVAs revealed a significant difference between participant race for feelings toward White people, $F(2, 779) = 20.13$, $p < .001$, $\eta^2 = .05$, 95% CI [0.02, 0.08], and People of color, $F(2, 793) = 15.37$, $p < .001$, $\eta^2 = .04$, 95% CI [0.02, 0.07], but not motivations to control prejudice, $F(2, 794) = 0.62$, $p < .54$. That is, White Americans reported higher positive feelings toward White people than did Asian and Latino/a/x Americans. Additionally, Latino/a/x Americans reported higher positive feelings toward people of color relative to Asian and White Americans (see Supplementary Table 3).

Primary ANOVA Analyses Using Full Sample

To assess the effects of race, gender, and emotional expression on evaluations of employees' competence, we conducted a 4 (applicant race: White, Black, Asian, Latino) by 2 (applicant gender: women, men) by 2 (emotion: sad, angry) ANOVA. Results revealed a main effect of emotion, $F(1, 914) = 31.98$, $p < .001$, $\eta_p^2 = .05$, 95% CI [0.01, 0.06]. Consistent with results in the manuscript, participants evaluated applicants as more competent if they expressed anger in response to being challenged (adjusted $M = 4.17$, $SE = .03$) than if they expressed sadness (adjusted $M = 3.93$, $SE = .03$). No significant effects were observed for employee race, $F(3, 914) = 0.67$, $p = .57$; gender, $F(1, 914) = 0.03$, $p = .87$, the race-by-gender interaction, $F(3, 914) = 0.84$, $p = .47$; the race-by-emotion interaction, $F(3, 914) = 1.86$, $p = .13$, the gender-by-emotion interaction, $F(1, 914) = 1.03$, $p = .31$, or the three-way interaction, $F(3, 914) = 0.37$, $p = .77$.

To test whether expressing anger would be detrimental for the workplace status of women but not men, we conducted a 2 (applicant gender: women, men) by 2 (emotion: sad, angry) ANOVA on standardized composite status scores. Results revealed a significant main effect for emotion, $F(1, 838) = 6.63$, $p = .01$, $\eta_p^2 = .01$, 95% CI [0.00, 0.02], such that angry employees (adjusted $M = -0.08$, $SE = .04$) were accorded less status than sad employees (adjusted $M = 0.05$, $SE = .04$). We also found a significant main effect of gender, $F(1, 838) = 5.43$, $p = .02$, $\eta_p^2 = .01$, 95% CI [0.00, 0.02], such that employees who were men (adjusted $M = 0.04$, $SE = .04$) were accorded more status than women (adjusted $M = -0.07$, $SE = .04$). These effects were qualified by a significant interaction between gender and emotion, which is displayed in Supplementary Figure 1, $F(1, 838) = 5.34$, $p = .02$, $\eta_p^2 = .01$, 95% CI [0.00, .02]. Bonferroni adjusted pairwise comparisons revealed that sad men (adjusted $M = .05$, $SE = .05$) and women (adjusted $M = .05$, $SE = .05$) were not accorded significantly different levels of status, $t(840) = 0.01$, $p = .99$, 95% CI [-0.14, 0.14]. However, angry women (adjusted $M = -0.20$, $SE = .05$) were accorded lower status than sad women, $t(840) = -3.46$, $p = .001$, 95% CI [-0.39, -0.11], sad men, $t(840) = -3.46$, $p = .001$, 95% CI [-0.39, -0.11], and angry men (adjusted $M = .04$, $SE = .05$; $t(840) = 3.29$, $p = .001$, 95% CI [0.09, 0.37].

We then tested whether evaluations of employees' status differed based on their race, gender, and emotion by conducting a 4x2x2 ANOVA. Results revealed significant main effects of race, $F(1, 826) = 2.86, p = .036, \eta_p^2 = .01$, 95% CI [0.00, 0.03], gender, $F(1, 826) = 5.71, p = .02, \eta_p^2 = .01$, 95% CI [0.00, 0.02], and emotion, $F(1, 826) = 6.39, p = .001, \eta_p^2 = .01$, 95% CI [0.01, 0.02]. Overall, women (adjusted M = -0.08, SE = .04) were accorded less status than men (adjusted M = 0.04, SE = .04), and people who expressed anger (adjusted M = -0.07, SE = .04) were accorded less status than people who expressed sadness (adjusted M = 0.05, SE = .04). Bonferroni corrected pairwise comparisons between applicants' race revealed that Black applicants (adjusted M = 0.10, SE = .05) were accorded more status than White applicants (adjusted M = -0.10, SE = .05; $t(840) = 2.84, p = .005$, 95% CI [0.06, 0.34]. The main effects were qualified by interactions between gender and emotion, $F(1, 836) = 5.49, p = .019, \eta_p^2 = .01$, 95% CI[0.00, 0.02] and race and emotion, $F(1, 826) = 3.89, p = .009, \eta_p^2 = .01$, 95% CI [0.00, 0.03]. The interaction between gender and emotion was consistent with the pattern displayed in Supplementary Figure 1. The interaction between race and emotion interaction showed that angry White employees (adjusted M = -0.26, SE = .07) were accorded the lowest status and angry Black employees (adjusted M = 0.17, SE = .07) were accorded the highest status (see Supplemental Figure 2). Bonferroni adjusted comparisons revealed that angry White employees were accorded less status than angry Black employees, sad White employees, (adjusted M = .06, SE = .07; $t(840) = -3.26, p = .001$, 95% CI [-0.52, -0.13], and sad Latino employees, (adjusted M = .08, SE = .07; $t(840) = 3.49, p = .001$, 95% CI [0.15, 0.53]. No significant effects were found for the gender-by-race interaction, $F(3, 826) = 2.23, p = .084$ or the three-way interaction, $F(3, 826) = 0.28, p = .84$.

Primary OLS Regression Analyses Using Full Sample

To assess the effects of race, gender, and emotional expression on evaluations of employees' composite competence, we conducted two ordinary least squares multiple regression models (see Supplementary Table 4). In Model 1, we regressed participant ratings of employee competence on employee race (Black, Asian, and Latino/a/x all in reference to White), gender (1 = men), expressed emotion (1 = angry) while controlling for mean-centered standardized composite status ratings, participant feelings toward White people and people of color, and motivation to control prejudice. Results revealed a main effect of emotion, $b = 0.26$, $t(814) = 6.41, p < .001$, 95% CI [0.18, 0.35], $\beta = .21$. In line with ANOVA models above and reported in the manuscript, participants evaluated employees as more competent if they expressed anger in response to being challenged than if they expressed sadness. In Model 2, we again regressed participant ratings of employee competence on employee race, gender, expressed emotion but included all combinations of the product term interactions between the variables and the three-way interactions between each racial group, gender, and emotion while controlling for mean-centered standardized composite status ratings, participant feelings toward White people and people of color, and motivation to control prejudice. Results revealed no significant effects for our primary variables of interest. Power analysis suggests that to detect a small effect ($f^2 = .02$) for a regression model with 19 terms requires a sample of 1,023 participants. Our model likely did not detect any effects given it only included 824 participants.

To test the effect that gender, race, and emotion expression had on status, we conducted 3 ordinary least squares multiple regression models (see Supplementary Table 5). In Model 1, we regressed standardized status on target race (all in reference to White), gender, and expressed

emotion while controlling for competence, feelings toward White people and people of color and motivation to control prejudice. Consistent with ANOVA results, we found main effects for Black targets, gender, and emotion expression. That is, Black employees were accorded more status relative to White employees, and women and people who expressed anger were accorded lower status. In Model 2, we included target gender, expressed emotion, and the product term interaction with all controls. Results revealed a significant main effect of emotion, such that employees who expressed anger were accorded less status than those who expressed sadness. The effect was qualified by a significant interaction where women who expressed anger were accorded the lower status than sad women, sad men, and angry men (see Supplementary Figure 2). In Model 3, we included employee race, gender, expressed emotion, and all combinations of two- and three-way interactions. This model yielded no significant effects among our primary variables of interest, which was likely due to insufficient sample size.

Supplementary Table 1

Correlations and Descriptive Statistics Among Study Variables

	1	2	3	4	5	6	7	8	9	10	11
1. Target gender (1 = men)	–										
	(930)										
2. Target emotion (1 = angry)	-.00	–									
	(930)	(930)									
3. Participant gender (1 = men)	.02	-.07	–								
	(891)	(891)	(891)								
4. Status (composite) [a]	.78*	-.09*	.08*	–							
	(842)	(842)	(842)	(842)							
5. Salary	.10**	-.09*	.05	.58***	–						
	(848)	(848)	(848)	(842)	(848)						
6. Status	.01	-.08*	.05	.83***	.27***	–					
	(927)	(927)	(888)	(842)	(845)	(927)					
7. Power	.06	-.08*	.06	.81***	.24***	.67***	–				
	(927)	(927)	(888)	(842)	(845)	(926)	(927)				
8. Independence	.04	-.02	.08*	.75***	.22***	.53***	.51***	–			
	(927)	(927)	(888)	(842)	(845)	(924)	(925)	(927)			
9. Competence (composite) [b]	.01	.18***	.04	.36***	.23***	.31***	.21***	.29***	–		
	(930)	(930)	(891)	(842)	(848)	(927)	(927)	(927)	(930)		
10. Capable	.03	.17***	.06	.31***	.20***	.27***	.18***	.25***	.90***	–	
	(930)	(930)	(891)	(842)	(848)	(927)	(927)	(927)	(930)	(930)	
11. Competent	-.02	.16***	.01	.34***	.21***	.28***	.20***	.27***	.91***	.63***	–
	(930)	(930)	(891)	(842)	(848)	(927)	(927)	(927)	(930)	(930)	(930)

	1	2	3	4	5	6	7	8	9	10	11	12	13
1. White target	–												
	(930)												
2. Black target	-.32***	–											
	(930)	(930)											
3. Asian target	-.31***	-.31***	–										
	(930)	(930)	(930)										
4. Latino/a/x target	-.32***	-.32***	-.31***	–									
	(930)	(930)	(930)	(930)									
5. Prejudice control	.06	-.07*	.02	-.02	–								
	(930)	(930)	(930)	(930)	(930)								
6. Status (composite) [a]	-.07*	.09*	-.03	.01	.04	–							
	(842)	(842)	(842)	(842)	(842)	(842)							
7. Salary	-.04	.07*	-.01	-.02	.01	.58***	–						
	(842)	(927)	(848)	(848)	(848)	(842)	(848)						
8. Status	-.09**	.09**	-.03	.02	.05	.83***	.27***	–					
	(927)	(927)	(927)	(927)	(927)	(842)	(845)	(927)					
9. Power	-.07*	.06	-.02	.03	.04	.81***	.24***	.67***	–				
	(927)	(927)	(927)	(927)	(927)	(842)	(845)	(926)	(927)				
10. Independence	-.02	.01	-.02	.01	.06	.75***	.22***	.53***	.51***	–			
	(927)	(927)	(927)	(927)	(927)	(842)	(845)	(924)	(925)	(927)			
11. Competence (composite) [b]	-.03	.04	-.03	.03	.11**	.36***	.23***	.31***	.21***	.29***	–		
	(930)	(930)	(930)	(930)	(930)	(842)	(848)	(927)	(927)	(927)	(930)		
12. Capable	-.01	.02	-.02	.03	.11***	.31***	.20***	.27***	.18***	.25***	.90***	–	
	(930)	(930)	(930)	(930)	(930)	(842)	(848)	(927)	(927)	(927)	(930)	(930)	
13. Competent	-.04	.04	-.03	.02	.08*	.34***	.21***	.28***	.20***	.27***	.91***	.63***	–
	(930)	(930)	(930)	(930)	(930)	(842)	(848)	(927)	(927)	(927)	(930)	(930)	(930)

	1	2	3	4	5	6	7	8	9	10	11	12	13
1. White participants	–												
	(891)												
2. Asian participants	-.36***	–											
	(891)	(891)											
3. Latino/a/x participants	-.26***	-.61***	–										
	(891)	(891)	(891)										
4. Feelings toward Whites	.20***	-.12	-.02	–									
	(874)	(874)	(874)	(911)									
5. Feelings toward POC	-.03	-.14	.18***	.57***	–								
	(890)	(890)	(890)	(910)	(929)								
6. Status (composite) [a]	.03	-.05	.03	.05	.01**	–							
	(842)	(842)	(842)	(825)	(841)	(842)							
7. Salary	.12***	-.09**	-.02	.06*	.07**	.58***	–						
	(848)	(848)	(848)	(831)	(847)	(842)	(848)						
8. Status	-.03	-.06	.07*	.02	.10**	.83***	.27***	–					
	(888)	(888)	(888)	(908)	(926)	(842)	(845)	(927)					
9. Power	-.00	-.02	.04	.04	.09**	.81***	.24***	.67***	–				
	(927)	(888)	(888)	(908)	(926)	(842)	(845)	(926)	(927)				
10. Independence	-.03	-.04	.03	.03	.10**	.75***	.22***	.53***	.51***	–			
	(888)	(888)	(888)	(908)	(926)	(842)	(845)	(924)	(925)	(927)			
11. Competence (composite) [b]	.08*	-.12***	.07*	.04	.11***	.36***	.23***	.31***	.21***	.29***	–		
	(891)	(891)	(891)	(911)	(929)	(842)	(848)	(927)	(927)	(927)	(930)		
12. Capable	.07*	-.10**	.06	.06	.11***	.31***	.20***	.27***	.18***	.25***	.90***	–	
	(891)	(891)	(891)	(911)	(929)	(842)	(848)	(927)	(927)	(927)	(930)	(930)	
13. Competent	.07*	-.11**	.07*	.01	.09**	.34***	.21***	.28***	.20***	.27***	.91***	.63***	–
	(891)	(891)	(891)	(911)	(930)	(842)	(848)	(927)	(927)	(927)	(930)	(930)	(930)

	1	2	3	4	5	6	7	8	9	10	11	12	13
1. Target gender (1 = men)	–												
	(930)												
2. Target emotion (1 = angry)	-.00	–											
	(930)	(930)											
3. Participant gender (1 = men)	.02	-.07***	–										
	(891)	(891)	(891)										
4. White target	-.01	.01	.05	–									
	(930)	(930)	(891)	(930)									
5. Black target	.01	-.00	-.04	-.32***	–								
	(930)	(930)	(891)	(930)	(930)								
6. Asian target	-.02	-.01	-.02	-.31***	-.31***	–							
	(930)	(930)	(891)	(930)	(930)	(930)							
7. Latino/a/x target	-.03	-.01	.01	-.32***	-.32***	.31***	–						
	(930)	(930)	(891)	(930)	(930)	(930)	(930)						
8. Prejudice control	.05	.06	-.20***	.06	-.07*	.02	-.02	–					
	(930)	(930)	(891)	(930)	(930)	(930)	(930)	(930)					
9. White participants	-.02	.01	-.00	-.01	.01	.01	-.01	-.02	–				
	(891)	(891)	(891)	(891)	(891)	(891)	(891)	(891)	(891)				
10. Asian participants	.01	-.01	.07*	.02	-.02	.00	-.01	.04	-.36***	–			
	(891)	(891)	(891)	(891)	(891)	(891)	(891)	(891)	(891)	(891)			
11. Latino/a/x participants	.02	.02	-.08*	.02	-.02	.02	.02	-.01	-.26***	-.61***	–		
	(891)	(891)	(891)	(891)	(891)	(891)	(891)	(891)	(891)	(891)	(891)		
12. Feelings toward Whites	-.02	-.03	.01	.03	.03	.00	-.05	.09*	.20***	-.12***	-.02	–	
	(911)	(911)	(874)	(911)	(911)	(911)	(911)	(911)	(874)	(874)	(874)	(911)	
13. Feelings toward POC	.02	-.01	-.01	.01	.00	.03	.01	.15***	-.03	-.14***	.18***	.57***	–
	(929)	(929)	(890)	(929)	(929)	(929)	(929)	(929)	(890)	(890)	(890)	(910)	(929)

Note. For ease of presentation, associations for covariates were paired with major dependent variables, so all associations between dependent variables are redundant in each panel. *n* is listed in parentheses beneath each correlation. Prejudice control = Motivation to Control Prejudice Reactions score. POC = people of color.
[a] Standardized composite of salary, status, power, and independence variables. [b] Composite of capable and competent variables.
* $p < .05$, ** $p < .01$, *** $p < .001$.

Supplementary Table 2

Means by Condition for Variables

	Competence [a]											
Group	White			Black			Asian			Latino/a/x		
	n	*M*	*SD*	*n*	*M*	*SD*	*n*	*M*	*SD*	*n*	*M*	*SD*
Angry women	60	4.10	0.57	57	4.25	0.61	57	4.04	0.61	58	4.19	0.55
Angry men	61	4.14	0.65	62	4.31	0.62	57	4.16	0.61	55	4.19	0.59
Sad women	56	3.97	0.72	56	3.80	0.80	58	3.93	0.75	58	4.08	0.67
Sad men	58	3.88	0.71	62	3.92	0.78	59	3.94	0.62	56	3.91	0.62
	Status [b]											
Group	White			Black			Asian			Latino/a/x		
	n	*M*	*SD*	*n*	*M*	*SD*	*n*	*M*	*SD*	*n*	*M*	*SD*
Angry women	53	-0.33	0.83	52	-0.08	0.67	51	-0.22	0.73	55	-0.16	0.88
Angry men	56	-0.20	0.67	53	0.42	0.67	52	-0.04	0.70	51	-0.02	0.73
Sad women	49	0.04	0.61	51	-0.07	0.70	55	0.09	0.81	56	0.13	0.77
Sad men	55	0.08	0.67	52	0.12	0.70	49	-0.03	0.52	52	0.03	0.64

[a] Composite of capable and competent variables (ranges from 1 to 5). [b] Standardized composite of salary, status, power, and independence variables.

Supplementary Table 3

Feeling Thermometer and Motivation to Control Prejudice Reactions Means by Participant Race

Variable	Participant Race								
	White			Asian			Latino/a/x		
	n	*M*	*SD*	*n*	*M*	*SD*	*n*	*M*	*SD*
Prejudice control [a]	120	4.37	0.78	406	4.42	0.60	271	4.40	0.64
Feelings toward Whites [b]	117	78.33	18.11	400	64.81	19.65	265	66.88	22.28
Feelings toward POC [b]	120	70.11	20.03	406	68.77	16.19	270	76.04	16.53

Note. Prejudice control = Motivation to Control Prejudice Reactions score.
[a] Variable ranges from 1 to 7. [b] Variable ranges from 0 to 100. Higher numbers indicate increases.

Supplementary Table 4

Composite Competence Scores Regressed on Race, Gender, and Emotion (N = 824)

Variable	Model 1			Model 2		
	b	*SE(b)*	*β*	*b*	*SE(b)*	*β*
Target race (0 = White)						
Black	0.02	0.06	.01	-0.15	0.12	.10
Asian	0.02	0.06	.01	0.00	0.12	.00
Latino	0.03	0.06	.02	0.04	0.12	.03
Target gender (1 = men)	-0.03	0.04	.02	-0.12	0.12	.09
Target emotion (1 = angry)	0.26***	0.04	.21	0.17	0.12	.13
Black x emotion				0.28	0.17	.14
Black x gender				0.21	0.17	.11
Asian x emotion				-0.03	0.17	.02
Asian x gender				0.10	0.17	.05
Latino x emotion				0.04	0.17	.02
Latino x gender				-0.02	0.17	.01
Gender x emotion				0.15	0.16	.10
Black x emotion x gender				-0.32	0.23	.12
Asian x emotion x gender				-0.08	0.23	.03
Latino x emotion x gender				-0.05	0.23	.02
Status [a,b]	0.32***	0.03	.37	0.32***	0.03	.36
Feelings toward POC [a]	0.00	0.00	.05	0.00	0.00	.06
Feelings toward Whites [a]	0.00	0.00	.01	0.00	0.00	.01
Prejudice control [a]	0.08*	0.03	.08	0.07*	0.03	.07
Constant	3.92***	0.05		3.97***	0.09	
F(df, df)	*F*(9, 814) = 20.00***			*F*(19, 804) = 9.75***		
Adjusted R^2	.17			.17		

Note. POC = people of color. Prejudice control = Motivation to Control Prejudice Reactions score.
[a] Variable is mean-centered. [b] Composite of standardized salary, status, power, and independence variables.
**p* < .05, ***p* < .01, ****p* < .001.

Supplementary Table 5

Standardized Status Regressed on Race, Gender, and Emotion (N = 824)

Variable	Model 1			Model 2			Model 3		
	b	*SE(b)*	*β*	*b*	*SE(b)*	*β*	*b*	*SE(b)*	*β*
Target race (0 = White)									
Black	0.19**	0.07	.11				-0.15	0.12	.10
Asian	0.06	0.07	.03				0.00	0.12	.00
Latino	0.08	0.07	.05				0.04	0.12	.03
Target gender (1 = men)	0.10*	0.05	.07	-0.01	0.07	.01	-0.12	0.12	.09
Target emotion (1 = angry)	-0.23***	0.05	.16	-0.34***	0.07	.23	0.17	0.12	.13
Gender x emotion				0.22*	0.09	.13	0.15	0.16	.10
Black x emotion							0.28	0.17	.14
Black x gender							0.21	0.17	.11
Asian x emotion							-0.03	0.17	.02
Asian x gender							0.10	0.17	.05
Latino x emotion							0.04	0.17	.02
Latino x gender							-0.02	0.17	.01
Black x emotion x gender							-0.32	0.23	.12
Asian x emotion x gender							-0.08	0.23	.03
Latino x emotion x gender							-0.05	0.23	.02
Competence [a,b]	0.42	0.04	.37	0.43	0.04	.37	0.32***	0.03	.36
Feelings toward POC [a]	0.00	0.00	.07	0.00	0.00	.08	0.00	0.00	.06
Feelings toward Whites [a]	-0.00	0.00	.02	-0.00	0.00	.01	0.00	0.00	.01
Prejudice control [a]	0.00	0.04	.00	-0.01	0.04	.01	0.07*	0.03	.07
Constant	-0.03	0.06		0.11*	0.05		3.97	0.09	
F(df, df)	$F(9, 814)$ =18.29***			$F(7, 816)$ = 23.08***			$F(19, 804)$ = 9.75***		
Adjusted R^2	.16			.16			.17		

Note. POC = people of color. Prejudice control = Motivation to Control Prejudice Reactions score.
[a] Variable is mean-centered. [b] Composite of capable and competent variables.
*p < .05, **p < .01, ***p < .001.
