# Supplementary figures and images for: Workplace Anger Costs Women Irrespective of Race
Source: Front Psychol. 2020 Nov 6;11:579884. doi: 10.3389/fpsyg.2020.579884 (PMC7677348; doi:10.3389/fpsyg.2020.579884)

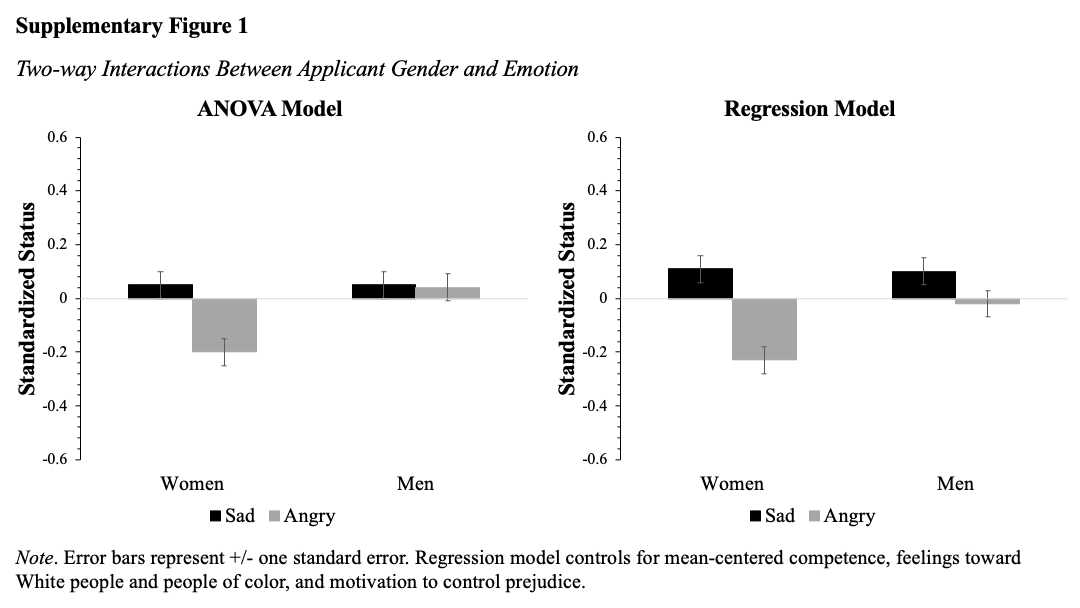

Supplement: Supplementary file 5 [file Image_1.TIFF]

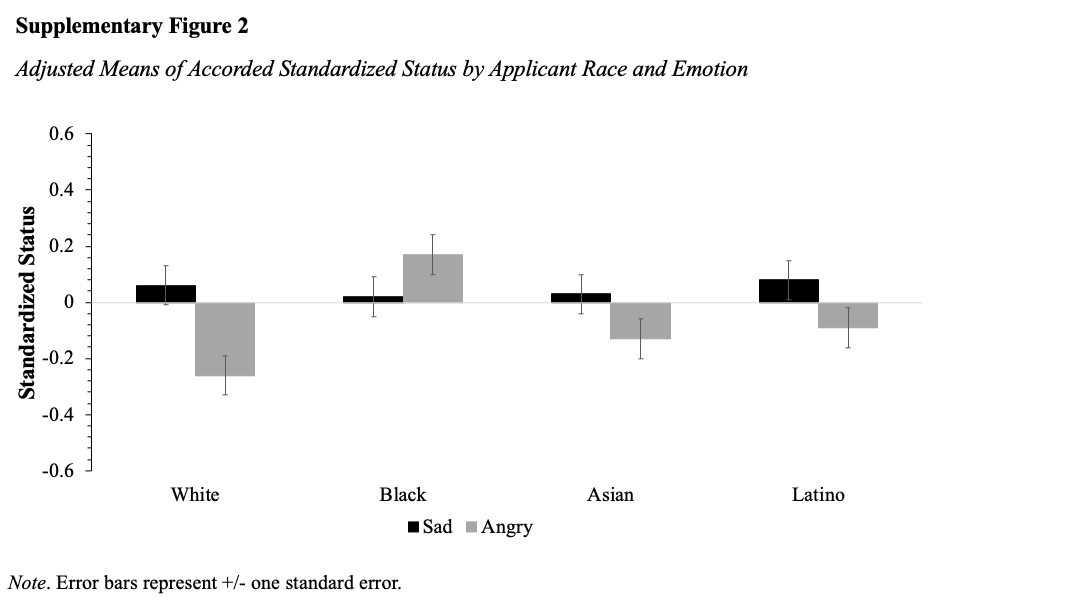

Supplement: Supplementary file 6 [file Image_2.TIFF]
